# Data in support of the identification of neuronal and astrocyte proteins interacting with extracellularly applied oligomeric and fibrillar α-synuclein assemblies by mass spectrometry

**DOI:** 10.1016/j.dib.2016.02.018

**Published:** 2016-02-12

**Authors:** Amulya Nidhi Shrivastava, Virginie Redeker, Nicolas Fritz, Laura Pieri, Leandro G. Almeida, Maria Spolidoro, Thomas Liebmann, Luc Bousset, Marianne Renner, Clément Léna, Anita Aperia, Ronald Melki, Antoine Triller

**Affiliations:** a École Normale Supérieure, Institut de Biologie de l’ENS (IBENS), INSERM, CNRS, PSL Research University, 46 Rue d׳Ulm, Paris 75005, France; b Paris-Saclay Institute of Neuroscience, CNRS, Gif-sur-Yvette 91198, France; c Department of Women and Children׳s Health, Karolinska Institutet, 171 76 Stockholm, Sweden

**Keywords:** Parkinson׳s disease, Alpha-synuclein assemblies, Proteomic Analysis, Pull down

## Abstract

α-Synuclein (α-syn) is the principal component of Lewy bodies, the pathophysiological hallmark of individuals affected by Parkinson disease (PD). This neuropathologic form of α-syn contributes to PD progression and propagation of α-syn assemblies between neurons. The data we present here support the proteomic analysis used to identify neuronal proteins that specifically interact with extracellularly applied oligomeric or fibrillar α-syn assemblies (conditions 1 and 2, respectively) (doi: 10.15252/embj.201591397[1]). α-syn assemblies and their cellular partner proteins were pulled down from neuronal cell lysed shortly after exposure to exogenous α-syn assemblies and the associated proteins were identified by mass spectrometry using a shotgun proteomic-based approach. We also performed experiments on pure cultures of astrocytes to identify astrocyte-specific proteins interacting with oligomeric or fibrillar α-syn (conditions 3 and 4, respectively). For each condition, proteins interacting selectively with α-syn assemblies were identified by comparison to proteins pulled-down from untreated cells used as controls. The mass spectrometry data, the database search and the peak lists have been deposited to the ProteomeXchange Consortium database via the PRIDE partner repository with the dataset identifiers *PRIDE:* PXD002256 to *PRIDE:* PXD002263 and doi: 10.6019/PXD002256 to 10.6019/PXD002263.

**Specifications Table**

**Table:** 

Subject area	*Biology*
More specific subject area	*Proteomic, Neurobiology, Neurodegenerative diseases*
Type of data	*Mass spectrometry data and tables with identified protein.*
How data was acquired	*Tryptic peptides were analyzed by nanoLC-MS/MS using an EASY-nLC HPLC system (Proxeon, Thermo) coupled to a LTQ-Orbitrap-velos (Thermo) mass spectrometer. Data acquisitions were conducted in the data-dependent acquisition mode.*
Data format	*.Raw files from the instrument, .dat peak list files, .mzid identification result files using MASCOT*
Experimental factors	*Cultured primary cortical neurons and astrocytes were exposed for 10 min to oligomeric or fibrillar S-tagged a-syn.*
Experimental features	*Neuronal or astrocyte partner proteins of a-syn oligomers and fibrils were purified with the a-syn assemblies on S-protein agarose beads. Proteins were digested on S-protein agarose beads using trypsin. Tryptic peptides were analyzed by nanoLC-MS/MS. Proteins were identified in the SwissProt database with MASCOT. Proteins interacting selectively with a-syn assemblies were identified by comparison to controls neurons and astrocytes that were not exposed to a-syn assemblies.*
Data source location	*Neuroscience Institute of Paris-Saclay, “Protein misfolding and aggregation in Neurodegenerative Diseases” team, CNRS, Gif-sur-Yvette, France.*
Data accessibility	*Data are within this article. The mass spectrometry proteomic data (neurons and astrocytes) have been deposited to the ProteomeXchange Consortium via the PRIDE partner repository with the dataset identifiers PRIDE: PXD002256 to PRIDE: PXD002263 and DOI 10.6019/PXD002256 to 10.6019/PXD002263. The list of identified proteins are presented in*Supplementary Tables 1–4.

**Value of the data**

• Data from a proteomic-based screen identify a list of proteins from neurons and astrocytes that interact with extracellularly applied oligomeric or fibrillar α-syn.
• The large dataset of proteins interacting with exogenous oligomeric and fibrillar α-syn includes several extracellularly exposed proteins, corresponding to transmembrane proteins, cell surface proteins and secreted proteins.
• The list of extracellularly exposed partner proteins identifies targets of exogenous α-syn assemblies, among which Na^+^/K^+^ ATPase subunit α3 is the only neuronal specific transmembrane protein interacting with both oligomeric and fibrillar α-syn.

## 1 Data

**Fig. 1:**
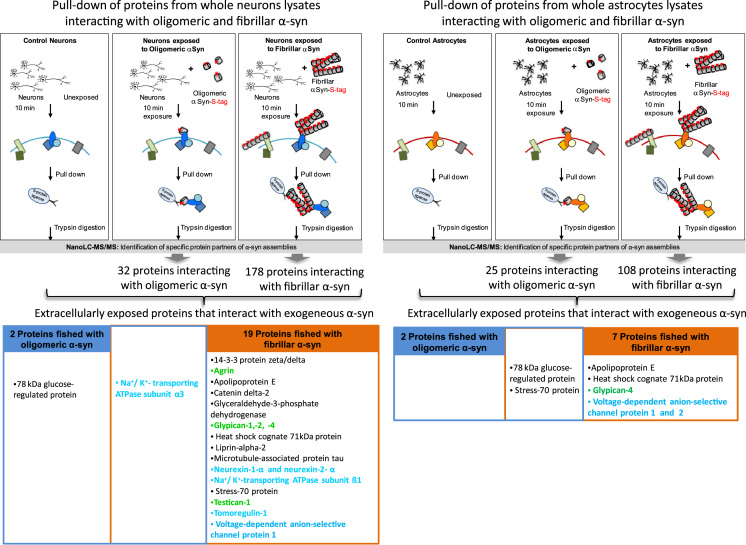
Experimental strategy for proteomic identification of proteins interacting specifically with extracellularly applied oligomeric and fibrillar α-syn. Rat cortical neurons (left panel) or astrocytes (right panel) were incubated for 10 min with oligomeric or fibrillar α-syn-S-tag (at a particle concentration of 1 µM and 4.8 nM, respectively, equivalent to 40 µM monomeric α-syn). After cell lysis, fresh protein extracts from those cells were incubated with S-protein agarose beads to pull down α-syn-S-tag assemblies together with their specific protein partners. Unexposed cell extracts incubated with S-protein agarose beads were used as a control. Proteins bound to the S-protein agarose beads were subjected to trypsin digestion and subsequently identified and quantified by nanoLC-MS/MS analysis, using a nanoLC-LTQ-Orbitrap. The complete list of identified proteins is shown in Supplementary Tables 1–4. Our data mining analysis highlighted a list of interacting protein partners of oligomeric (blue frame) or fibrillar α-syn (orange frame) described as extracellularly exposed in peer-reviewed articles: in blue transmembrane proteins with extracellular domains, in green proteoglycans and in black proteins that can be secreted or extracellularly localized.

Fibrillar α-Synuclein (α-syn) is the principal component of Lewy bodies, the pathophysiological hallmark of individuals affected by Parkinson disease (PD). This neuropathologic form of α-syn contributes to PD progression and propagation of α-syn assemblies between neurons. The aim of this study [1] was to identify membrane proteins interacting with extracellularly applied α-syn assemblies and exposed at the surface of neurons using a proteomic-based approach (Fig. 1) [1]. Data from this proteomic-based screen performed on neurons and astrocytes cultures give a list of neuronal and astrocytic proteins that interact with extracellularly applied oligomeric or fibrillar α-syn. Data mining of this large dataset of proteins highlights extracellularly exposed proteins interacting with exogenous oligomeric and/or fibrillar α-syn.

## 2 Experimental design, materials and methods

### 2.1 Experimental design

The data we present here support this proteomic analysis performed to identify neuronal membrane proteins that specifically interact with extracellularly applied oligomeric or fibrillar α-syn assemblies (Fig. 1). Therefore, recombinant α-syn (oligomeric and fibrillar forms) with a C-terminal S-tag that binds with high affinity to ribonuclease S-protein [2] were used to identify specific partners of extracellularly applied α-syn in neuronal cells. Two weeks old pure cultures of cortical neurons were exposed to oligomeric or fibrillar S-tagged α-syn for 10 min (conditions 1 and 2 respectively). α-syn-S-tag assemblies and associated proteins were pulled down from whole-cell lysates using S-protein agarose beads, trypsin digested, and the resulting peptides were identified by nanoLC-MS/MS. Control samples were prepared for each condition from neurons unexposed to α-syn. Protein abundance was assessed by a label-free quantitative proteomic method using spectral counting. From whole neuron lysates, we identified 32 and 178 protein partners for oligomeric and fibrillar α-syn, respectively (Supplementary Tables 1 and 2). Several intracellular proteins were identified in the screen due to the interaction of α-syn with cytosolic partners following their endocytosis [3], [4], [5] and/or interaction following cell disruption during protein extraction. As pure cultures of neurons were maintained in astrocyte-conditioned neuronal medium and in order to discriminate between astrocyte and neuron specific proteins, we made a control Pull down/MS experiment in which pure cultures of astrocytes were exposed to oligomeric and fibrillar S-tag α-syn (conditions 3 and 4 respectively). From whole astrocyte lysates, we identified 25 and 108 protein partners for oligomeric and fibrillar α-syn, respectively (Supplementary Tables 3 and 4 respectively). The list of extracellularly exposed partner proteins identified targets of exogenous α-syn assemblies, among which Na^+^/K^+^ ATPase subunit α3 was the only neuronal specific transmembrane protein interacting with both oligomeric and fibrillar α-syn (Fig. 1). The proteomic data have been deposited to the ProteomeXchange Consortium database [6] via the PRIDE partner repository with the dataset identifiers PRIDE:PXD002256 to PRIDE:PXD002263, and are associated with a research article published in EMBO J [1].

### 2.2 Preparation and characterization of α-syn-S-tag assemblies

C-terminally S-tagged (α-syn-S-tag) human α-syn was expressed and purified as described previously [7]. α-syn concentration was determined spectrophotometrically using an extinction coefficient of 5960 M^−1^cm^−1^ at 280 nm. Pure monomeric α-syn (0.2–0.5 mM) in 50 mM Tris–HCl, pH 7.5, 150 mM KCl was filtered through sterile 0.22 µm filters and stored at −80 °C. For oligomers and fibrils formation, monomeric α-syn in buffer A were incubated at 4 °C for 7 days or 37 °C for 4 days under continuous shaking in a thermomixer (Eppendorf, Germany) set at 600 rpm, respectively. Assembly into fibrils was monitored using Thioflavin T binding. Aliquots (10 µl) were withdrawn at different time intervals from the assembly reaction and mixed with 400 µl of Thioflavin T (10 µM) in water and Thioflavin T fluorescence (Excitation wavelength: 440 nm and emission wavelength: 480 nm) was recorded using a Cary Eclipse Spectrofluorometer (Varian Inc., Palo Alto, USA). Oligomeric α-syn was separated from monomeric α-syn by size exclusion chromatography using a Superose^®^6 HR10/30 column (GE Healthcare) equilibrated in phosphate buffered saline (PBS) buffer. Fibrillar α-syn was separated from monomeric α-syn through 2-cycles of sedimentation at 15,000*g* and re-suspension of the pellet.

The nature of all α-syn assemblies used was assessed using a Jeol 1400 (Jeol Ltd., Peabody, MA) Transmission Electron Microscope (TEM) after adsorption of the samples onto carbon-coated 200-mesh grids and negative staining with 1% uranyl acetate. The images were acquired with a GatanOrius CCD camera (Gatan). The particle concentration of oligomeric and fibrillar α-syn samples was assessed by analytical ultracentrifugation (AUC) and quantitative transmission electron microscopy (TEM) as previously described [8]. The particle concentration of α-syn (tagged and untagged) was obtained by dividing α-syn monomeric concentration by the average number of molecules (as measured by AUC and TEM). For our preparation, the average number of molecules measured for oligomeric and fibrillar α-syn was 40 and 8333, respectively. The particles concentrations for oligomeric α-syn was 25 nM and that for fibrillar α-syn was 0.03 nM, unless specified, corresponding to 1 µM or 0.25 µM monomeric α-syn, respectively.

### 2.3 Primary neuronal and astrocyte cultures and exposure to α-syn-S-tag assemblies

All cultures were prepared from 18-day-old Sprague-Dawley rat embryos (Janvier Labs, France). Pull down and proteomic studies were performed on two weeks old pure cultures of rat cortical neurons (DIV14) plated on 10 cm plates pre-coated with 80 mg/ml poly-D,L-ornithine. Cortical neurons were used, as they can be prepared in larger quantities (4×10^6^ cells/dish) as required for these proteomic experiments. Freshly dissociated (trypsin) cortices were plated (4×10^6^ cells per 10 cm dish) in neuronal attachment media consisting of 10% horse serum, 1 mM sodium pyruvate (Life Technologies), and 2 mM glutamine (Life Technologies) in minimum essential medium (MEM) (Life Technologies) for 3 h. Pure neuronal cultures were maintained in astrocyte-conditioned neuronal medium (see below) supplemented with cytosine-arabinoside (5 µM) as recently described [9].

Primary astrocyte cultures from rat cortex were prepared as described recently [9]. The culture medium consisted of MEM supplemented with fetal bovine serum (10%, PAA Labs), sodium pyruvate (1 mM), glutamine (2 mM), and penicillin/streptomycin (Life Technologies). The medium was replaced after 24 h with fresh media.

Astrocyte cultures for feeding pure neurons: Astrocytes were plated and cultured in 10 cm dishes as described above. At 10 DIV, cytosine-arabinoside containing media was added for 48 h. The medium was then replaced with neuronal media containing: neurobasal medium, penicillin/streptomycin, B27 (13, Life Technologies), glutamine (2 mM), and horse serum (5%, PAA Labs). Every other day, this astrocyte-conditioned medium was used to feed neurons, and fresh neuronal medium was added to the astrocyte cultures [9].

Recombinant oligomeric or fibrillar α-syn-S-tag (40 µM monomer concentration) were added to the culture medium of 2 weeks old pure cortical neuron cultures of rat (conditions 1 and 2 respectively; 3–4 culture dishes per condition). For each condition, unexposed neurons were used as control. After 10 minutes, cells were washed twice with 1X PBS and scraped on ice in 50 mM Hepes-KOH (pH 7.5), 2 mM EDTA, 0.1% Triton X-100, supplemented with complete protease inhibitor cocktail (Roche). The extracts were flash frozen in liquid nitrogen and stored at −80 °C. In order to discriminate between neuronal specific proteins and proteins from astrocytes, we made a control experiment in which pure astrocytes were exposed to recombinant S-tag α-syn assemblies (oligomeric and fibrillar forms in conditions 3 and 4 respectively). For each condition, control samples were prepared from pure astroctyes unexposed to α-syn. Three experimental replicates were performed for each condition and corresponding unexposed cell controls.

### 2.4 Pull down of α-syn-S-tag bound protein complexes and sample preparation for mass spectrometry.

Cell lysis was completed by sonication and the protein concentration in the extracts determined using BCA assay kit (Thermo Scientific). To pull down oligomeric or fibrillar α-syn-S-tag together with their specific protein partners, 0.5 mg of total protein extracts were incubated with S-protein agarose (200 µl settled resin) (Novagen) equilibrated in 500 µl binding buffer (20 mM Tris–HCl pH 7.5, 150 mM NaCl, 0.1% Triton X-100, Complete protease inhibitors) for 1 h at 4 °C under gentle agitation. Extracts from control-unexposed neurons were also incubated with S-protein agarose beads and used as control. After 3 washes with 5 ml binding buffer and 3 washes with 5 ml Triton-free binding buffer, the resin was re-suspended in 400 µl of 50 mM ammonium bicarbonate pH 8 in the presence of 0.1% RapiGest (Waters corporation, Milford, MA) and heated at 95 °C for 10 min. Proteins were then reduced in the presence of 10 mM dithiotreitol (DTT) at 56 °C for 30 min and alkylated in 20 mM iodoacetamide (Sigma) at room temperature in the dark for 45 min. Proteins bound to the S-protein agarose beads were digested on the resin by incubating the samples overnight at 37 °C in the presence of 0.8 µg trypsin Promega Gold (Promega, Madison, WI). After digestion, the samples were centrifuged for 10 min at 16,000*g* to discard the resins. Trypsin digestion and RapiGest treatment in the supernatants were stopped by addition of 0.5% TFA and incubation at 37 °C for 45 min. The tryptic peptide samples were spun for 10 min at 16,000*g* and the supernatants were stored at −80 °C for MS analysis.

### 2.5 Mass spectrometric analysis of the pulled down proteins

For each pull down, 15 µl of tryptic peptide digests were analyzed by nanoLC-MS/MS using an EASY-nLC II high performance liquid chromatography (HPLC) system (Proxeon, Thermo-Scientific, Waltham, MA) coupled to the nanoelectrospray ion source of a Linear Ion Trap-OrbitrapVelos mass spectrometer (Thermo Scientific). Peptide separation was performed on a reversed phase C18 nano HPLC column (100 µm inner diameter, 5 µm C18 particles, 15 cm length, NTCC-360/100-5) from Nikkyo Technos (Nikkyo Technos Co., Ltd., Tokyo, Japan). The peptides were loaded at a pressure-dependent flow rate corresponding to a maximum pressure of 200 bars and eluted at a flow rate of 300 nl/min using a two slope gradient of first 5 to 20% solvent B for 60 min, followed by 20% to 40% solvent B in 40 min and a washing step at 100% solvent B. Solvent A was 0.1% formic acid in water, and solvent B was 0.1% formic acid in 100% acetonitrile. NanoLC-MS/MS experiments were conducted in the data-dependent acquisition mode. The mass of the precursors was measured with a high resolution (60,000 full weight at half maximum) in the Orbitrap. The 20 most intense ions, above an intensity threshold of 5000 counts, were selected for CID fragmentation and analysis in the LTQ.

### 2.6 Data analysis

NanoLC-MS/MS raw data were processed automatically using the Scaffold software (version 3.6.4) and the SwissProt_18112011 database with both the Mascot [10] (Version: 2.3.02) and the X! Tandem [11] (Version CYCLONE 2010.12.01.1) search engine, a specific trypsin digestion with up to 2 missed cleavages, a tolerance of 0.5 Da for fragment monoisotopic masses and 6 ppm for parent monoisotopic mass tolerance, and the following chemical modifications: carbamidomethylation of Cys as fixed modification, and dehydration, ammonia loss, oxidation of methionine and N-acetylation as variable modifications. Scaffold (version Scaffold_3.6.4, Proteome Software Inc., Portland, OR) was used to validate MS/MS based peptide and protein identifications. Peptide identifications were accepted if they could be established at greater than 90.0% probability as specified by the Peptide Prophet algorithm [12]. Protein identifications were accepted if they could be established at greater than 99.0% probability and contained at least 2 identified peptides. Protein probabilities were assigned by the Protein Prophet algorithm [13]. Proteins that contained similar peptides and could not be differentiated based on MS/MS analysis alone were grouped to satisfy the principles of parsimony.

Identified proteins were quantified by a label-free proteomic approach using spectral counting [14] with the Scaffold software. Comparison between controls and samples identified some proteins as 60S ribosomal proteins, nucleolin and ribonuclease inhibitor as major contaminants. Among them nucleolin presented the best reproducibility among experiments and was chosen to normalize the spectral counting data. Only proteins identified with at least 2 unique peptides in at least 2 replicates were quantified. Only proteins with a spectral count ratio, between the cells exposed to α-syn (either oligomeric or fibrillar) and the control cells, above 1.6 and a *p*-value<0.05 were considered as significantly increased in the pull down and thus considered as α-syn interacting proteins. Spectral count ratios presented in Supplementary Tables 1–4 were calculated from averaged spectral counts of three independent replicates.

Several intracellular proteins were identified in the screen due to the interaction following endocytosis of α-syn assemblies [3], [4], [5] and/or interaction following cell disruption during protein extraction and processing. Among the identified candidates, we picked the proteins annotated as cell components of membrane, plasma membrane and/or extracellular regions, using the NCBI (National Center for Biotechnology Information) annotation tool of the Scaffold software based on Gene Ontology (GO) cell component term annotations [15] (Supplementary Tables 1–4). With this list of proteins, we performed a second search in Pubmed in order to select proteins for which we found an experimental description of extracellular exposure in peer-reviewed publications [16], [17], [18], [19], [20], [21], [22], [23], [24], [25], [26], [27], [28], [29], [30], [31] (Supplementary Tables 1–4). Only these latter proteins extracellularly exposed are reported in Fig. 1. This pull down data mining analysis identified several targets of exogenous assemblies α-syn, and indicated that Na^+^/K^+^ ATPase subunit α3 is the only transmembrane protein of our list with extracellularly exposed domains that was identified both with oligomeric and fibrillar α-syn (Fig. 1).

### 2.7 Proteomic data deposition

The mass spectrometry proteomic data (neurons and astrocytes) have been deposited to the ProteomeXchange Consortium [6] via the PRIDE partner repository with the dataset identifiers PXD002256 through PXD002263 and DOI 10.6019/PXD002256 through 10.6019/PXD002263.

## Footnotes

Appendix A Supplementary data associated with this article can be found in the online version at doi:10.1016/j.dib.2016.02.018.
