# Supplementary material for: Data in support of the identification of neuronal and astrocyte proteins interacting with extracellularly applied oligomeric and fibrillar α-synuclein assemblies by mass spectrometry
Source: Data Brief. 2016 Feb 12;7:221–8. doi: 10.1016/j.dib.2016.02.018 (PMC4773484; doi:10.1016/j.dib.2016.02.018)
Supplement: Supplementary file 2 — Supplementary material [file mmc2.pdf]

Table 1

List of proteins from whole neurons lysates interacting with extracellularly applied oligomeric α-syn

Fold change corresponds to the average spectral count ratio between exposed cells and control cells of three independent replicates. ∞: the spectral count ratio is infinite as the protein is pulled-down only with oligomeric α-synuclein. Membrane and/or extracellular proteins are indicated as determined using the NCBI annotation tool of the Scaffold software based on Gene Ontology (GO) cell component term annotations. References describing extracellular exposure in peer-reviewed articles are indicated. **In bold**, the unique plasma membrane protein pulled-down both with oligomeric and fibrillar α-synuclein and presenting both transmembrane and extracellularly exposed regions.

				GO cell component term annotations from NCBI			Experimental description of extracellular exposure in peer-reviewed articles
**Protein Name**	**Gene Name**	**Accession Number**	**Fold Change (Syn/Ctrl)**	**Membrane**	**Plasma membrane**	**Extracellular region**	
40S ribosomal protein S28	Rps28	P62858	7.0			+	
40S ribosomal protein S5	Rps5	P24050	2.2				
78 kDa glucose-regulated protein	Hspa5	P06761	5.4	+	+	+	+ [16,17]
AP-3 complex subunit delta-1	Ap3d1	O54774	1.8	+			
ATP synthase subunit beta, mitochondrial	Atp5b	P10719	2.0	+			
Calcium/calmodulin-dependent protein kinase type II subunit gamma	Camk2g	P11730	2.1	+			
Calmodulin	Calm1	P62161	2.5	+	+	+	
Creatine kinase B-type	Ckb	P07335	5.0	+	+	+	
Cytoskeleton-associated protein 5	Ckap5	A2AGT5	1.7	+			
Dihydropyrimidinase-related protein 3	Dpysl3	Q62188	2.1			+	
Double-stranded RNA-binding protein Staufen homolog 2	Stau2	Q68SB1	1.6				
Elongation factor 2	Eef2	P05197	2.0	+		+	
Elongation factor Tu, mitochondrial	Tufm	P85834	5.0	+		+	
ERC protein 2	Erc2	Q8K3M6	3.5	+	+		
Fragile X mental retardation syndrome-related protein 1	Fxr1	Q61584	2.0	+			
Friend of PRMT1 protein	Fop	Q9CY57	2.0				
GMP synthase [glutamine-hydrolyzing]	Gmps	Q3THK7	2.0				
Heterogeneous nuclear ribonucleoprotein K	Hnrnpk	P61979	1.8	+	+	+	
Kinesin-like protein KIF2A	Kif2a	P28740	1.6	+			
Myb-binding protein 1A	Mybbp1a	O35821	1.9	+			
Neuron navigator 1	Nav1	Q8CH77	1.6				
Non-POU domain-containing octamer-binding protein	Nono	Q5FVM4	1.7	+			
Protein unc-13 homolog A	Unc13a	Q62768	3.5	+	+		
Protein-L-isoaspartate(D-aspartate) O-methyltransferase	Pcmt1	P22062	2.5				
Serine/arginine-rich splicing factor 2	Srsf2	Q62093	2.4			+	
Serine/arginine-rich splicing factor 3	Srsf3	P84104	1.6				
SLIT-ROBO Rho GTPase-activating protein 2	Srgap2	Q91Z67	2.5	+		+	
**Sodium/potassium-transporting ATPase subunit alpha-3**	**Atp1a3**	**P06687**	**1.6**	**+**	**+**		+ [18]
Tubulin beta-4B chain	Tubb4b	P68372	1.6	+	+	+	
Ubiquitin carboxyl-terminal hydrolase isozyme L1	Uchl1	Q00981	∝	+	+	+	
Vesicle-associated membrane protein-associated protein A	Vapa	Q9WV55	4.0		+	+	
WD repeat-containing protein 47	Wdr47	Q8CGF6	2.8				

Table 2

List of proteins from whole neurons lysates interacting with extracellularly applied fibrillar α-syn

Fold change corresponds to the average spectral count ratio between exposed cells and control cells of three independent replicates. ∞: the spectral count ratio is infinite as the protein is pulled-down only with fibrillar α-synuclein.

Membrane and/or extracellular proteins are indicated as determined using the NCBI annotation tool of the Scaffold software based on Gene Ontology (GO) cell component term annotations. References describing extracellular exposure in peer-reviewed articles are indicated. **In bold**, the unique plasma membrane pulled-down both with oligomeric and fibrillar α-synuclein, and presenting both transmembrane and extracellularly exposed regions.

				GO cell component term annotations from NCBI			Experimental description of extracellular exposure in peer-reviewed articles
Protein name	Gene name	Accession Number	Fold Change (Syn/Ctrl)	Membrane	Plasma membrane	Extracellular region	
14-3-3 protein gamma	Ywhag	P61982	∝	+	+	+	
14-3-3 protein zeta/delta	Ywhaz	P63102	∝	+	+	+	+ [19]
40S ribosomal protein S15	RPS15	P62842	∝				
40S ribosomal protein S16	Rps16	P14131	3.0	+	+	+	
40S ribosomal protein S17	Rps17	P04644	5.0				
40S ribosomal protein S18	Rps18	P62270	6.7	+	+	+	
40S ribosomal protein S19	Rps19	P17074	∝				
40S ribosomal protein S20	Rps20	P60867	1.6	+		+	
40S ribosomal protein S25	Rps25	P62852	13.0			+	
40S ribosomal protein S29	Rps29	P62274	5.5	+	+	+	
40S ribosomal protein S5	Rps5	P24050	9.0				
40S ribosomal protein S7	Rps7	P62082	3.9	+	+	+	
5-azacytidine-induced protein 1	Azi1	Q62036	∝	+		+	
60S ribosomal protein L22-like 1	Rpl22l1	Q9D7S7	∝				
60S ribosomal protein L23	Rpl23	P62830	3.1	+	+	+	
60S ribosomal protein L27	Rpl27	P61354	2.1	+	+	+	
60S ribosomal protein L27a	Rpl27a	P18445	2.2				
60S ribosomal protein L38	Rpl38	P63174	∝				
Abl interactor 1	Abi1	Q8CBW3	18.0	+	+	+	
Acetyl-CoA acetyltransferase, mitochondrial	Acat1	P17764	∝	+		+	
Actin filament-associated protein 1	Afap1	Q8VH46	4.3				
Adenomatous polyposis coli protein	Apc	P70478	∝	+	+		
Adenomatous polyposis coli protein 2	Apc2	Q9Z1K7	∝	+	+		
ADP/ATP translocase 2	Slc25a5	P51881	3.7	+	+	+	
ADP-ribosylation factor GTPase-activating protein 3	Arfgap3	Q4KLN7	∝	+			
Agrin	Agrn	P25304	∝	+	+	+	+ [20]
Alpha-internexin	Ina	P23565	34.0				
Alpha-tubulin N-acetyltransferase	Atat1	Q6MG11	∝	+	+		
Amphiphysin	Amph	O08838	∝	+	+		
AP-3 complex subunit beta-2	Ap3b2	Q9JME5	∝	+			
AP-3 complex subunit delta-1	Ap3d1	O54774	4.3	+			
Apolipoprotein E	Apoe	P02650	∝	+	+	+	+ [21]
Ataxin-2	Atxn2	O70305	∝	+			
Ataxin-2-like protein	Atxn2l	Q7TQH0	∝	+			
ATP synthase subunit alpha, mitochondrial	Atp5a1	P15999	1.8	+	+		
ATP synthase subunit d, mitochondrial	Atp5h	P31399	∝	+			
ATP synthase subunit gamma, mitochondrial	Atp5c1	P35435	25.0	+			
ATP synthase subunit O, mitochondrial	Atp5o	Q06647	∝	+			
ATPase family AAA domain-containing protein 3	Atad3	Q3KRE0	∝	+			
ATP-dependent RNA helicase DDX3X	Ddx3x	Q62167	2.4	+		+	
Bcl-2-associated transcription factor 1	Bclaf1	Q8K019	10.5				
Calcium-binding mitochondrial carrier protein Aralar1	Slc25a12	Q8BH59	∝	+	+		
Calmodulin-regulated spectrin-associated protein 2	Camsap2	Q8C1B1	∝				
Calmodulin-regulated spectrin-associated protein 3	Camsap3	Q80VC9	∝	+	+		
CaM kinase-like vesicle-associated protein	Camkv	Q63092	6.0	+	+		
CAP-Gly domain-containing linker protein 2	Clip2	O55156	∝				
Caskin-1	Caskin1	Q8VHK2	∝				
Catenin delta-2	Ctnnd2	O35927	∝	+	+		+ [22]
Cell division control protein 42 homolog	Cdc42	P60766	∝	+	+	+	
Centrosomal protein of 170 kDa	Cep170	Q6A065	37.0	+			
Charged multivesicular body protein 2b	Chmp2b	Q8BJF9	∝	+		+	
Chromatin target of PRMT1 protein	Chtop	Q9CY57	5.3				
CLIP-associating protein 2	Clasp2	Q99JD4	1.7	+	+		
Coatomer subunit alpha	Copa	Q8CIE6	3.0	+		+	

Protein name	Gene name	Accession Number	Fold Change (Syn/Ctrl)	GO cell component term annotations from NCBI			Experimental description of extracellular exposure in peer-reviewed articles
				Membrane	Plasma membrane	Extracellular region	
Cofilin-1	Cfl1	P45592	3.8	+	+	+	
Cytoplasmic dynein 1 heavy chain 1	Dync1h1	Q9JHU4	62.0	+		+	
Dihydropyrimidinase-related protein 1	Crmp1	Q62950	4.5				
Dihydropyrimidinase-related protein 2	Dpysl2	O08553	1.6	+	+	+	
Disks large-associated protein 1	Dlgap1	Q9D415	∝	+		+	
Disks large-associated protein 4	Dlgap4	P97839	∝	+		+	
DnaJ homolog subfamily A member 1	Dnaja1	P63036	∝	+		+	
DnaJ homolog subfamily B member 1	Dnajb1	Q9QYJ3	∝			+	
DnaJ homolog subfamily B member 2	Dnajb2	Q9QYI5	∝				
DnaJ homolog subfamily B member 5	Dnajb5	O89114	∝				
DnaJ homolog subfamily B member 6	Dnajb6	O54946	∝	+			
Dynactin subunit 1	Dctn1	P28023	4.0				
Elongation factor 1-alpha 1	Eef1a1	P10126	2.6	+	+	+	
Elongation factor Tu, mitochondrial	Tufm	P85834	27.5	+		+	
Ena/VASP-like protein	Evl	O08719	∝	+	+		
Eukaryotic translation initiation factor 3 subunit C	Eif3c	B5DFC8	∝				
Glyceraldehyde-3-phosphate dehydrogenase	Gapdh	P04797	2.6				+ [23]
Glypican-1	Gpc1	P35053	∝	+	+	+	+ [24]
Glypican-2	Gpc2	P51653	∝	+	+	+	+ [24]
Glypican-4	Gpc4	P51655	∝	+	+	+	+ [24]
Guanine nucleotide-binding protein G(I)/G(S)/G(T) subunit beta-1	Gnb1	P54311	∝	+	+	+	
Heat shock cognate 71 kDa protein	HSPA8	P63018	5.0	+	+		+ [16]
Hepatoma-derived growth factor-related protein 2	Hdgfrp2	Q925G1	∝				
Heterochromatin protein 1-binding protein 3	Hp1bp3	Q6P747	6.0				
Heterogeneous nuclear ribonucleoprotein D0	Hnrnpd	Q60668	∝			+	
Heterogeneous nuclear ribonucleoprotein D-like	Hnrpdl	Q9Z130	∝			+	
Heterogeneous nuclear ribonucleoprotein K	Hnrnpk	P61979	4.5	+	+	+	
Heterogeneous nuclear ribonucleoprotein M	Hnrnpm	Q62826	5.4				
Heterogeneous nuclear ribonucleoprotein U-like protein 2	Hnrnpul2	Q00PI9	2.7	+			
Histone H2A.J	H2afj	A9UMV8	1.7				
Histone H2A.Z	H2afz	P0C0S6	6.0			+	
Histone H4	Hist1h4b	P62804	1.6	+		+	
Host cell factor 1	Hcfc1	Q61191	∝	+			
IQ motif and SEC7 domain-containing protein 1	Iqsec1	Q8R0S2	∝	+		+	
Kelch-like protein 22	Klhl22	D3ZZC3	∝				
KH domain-containing, RNA-binding, signal transduction-associated protein 1	Khdrbs1	Q91V33	∝	+			
Kinesin heavy chain isoform 5C	Kif5c	P28738	26.5				
Lamin-B1	Lmnb1	P70615	∝	+			
Lipid phosphate phosphatase-related protein type 4	Lppr4	Q7TME0	∝	+		+	
Liprin-alpha-2	Ppfia2	Q8BSS9	6.0			+	+ [25]
MAP/microtubule affinity-regulating kinase 3	Mark3	Q8VHF0	11.0	+	+		
MAP/microtubule affinity-regulating kinase 4	Mark4	Q8CIP4	∝				
MAP7 domain-containing protein 1	Map7d1	A2AJI0	∝				
Microtubule-associated protein 1B	Map1b	P14873	2.7	+	+		
Microtubule-associated protein 1S	Map1s	P0C5W1	∝	+	+		
Microtubule-associated protein 2	Map2	P15146	5.9				
Microtubule-associated protein 4	Map4	Q5M7W5	3.5	+		+	
Microtubule-associated protein 6	Map6	Q63560	3.0				
Microtubule-associated protein tau	Mapt	P19332	5.4	+	+		+ [26]
Microtubule-associated serine/threonine-protein kinase 1	Mast1	Q810W7	∝	+	+		
Mitochondrial glutamate carrier 1	Slc25a22	Q9D6M3	∝	+			
Mitogen-activated protein kinase kinase kinase kinase 4	Map4k4	P97820	∝				
Myc box-dependent-interacting protein 1	Bin1	O08539	∝	+	+		
Myosin regulatory light chain 12B	Myl12b	P18666	∝				
Nck-associated protein 1	Nckap1	P28660	4.0	+	+	+	
Neurexin-1-alpha	Nrxn1	Q63372	∝	+	+		+ [27]
Neurexin-2-alpha	Nrxn2	Q63374	∝	+			+ [28]
Neurofilament light polypeptide	Nefl	P19527	∝				
Neurofilament medium polypeptide	Nefm	P12839	∝				
Neuromodulin	Gap43	P07936	4.0	+	+		
Neuron navigator 1	Nav1	Q8CH77	44.3				

Protein name	Gene name	Accession Number	Fold Change (Syn/Ctrl)	GO cell component term annotations from NCBI			Experimental description of extracellular exposure in peer-reviewed articles
				Membrane	Plasma membrane	Extracellular region	
Neuron navigator 3	Nav3	Q80TN7	∝	+			
Neuronal migration protein doublecortin	Dcx	O88809	4.2				
Nucleolar and coiled-body phosphoprotein 1	Nolc1	P41777	∝				
Pinin	Pnn	O35691	∝	+	+		
Polymerase delta-interacting protein 3	Poldip3	Q8BG81	∝				
Prelamin-A/C	Lmna	P48679	∝	+			
Prickle-like protein 2	Prickle2	Q80Y24	∝	+	+		
Prohibitin-2	Phb2	O35129	∝	+		+	
Protein bassoon	Bsn	O88778	11.0	+	+		
Protein FAM54B	Fam54b	Q5XII9	∝				
Protein Hook homolog 3	Hook3	Q8BUK6	∝	+			
Protein KIAA0284	Kiaa0284	Q80U49	∝				
Protein NipSnap homolog 1	Nipsnap1	O55125	∝	+			
Protein piccolo	Pclo	Q9JKS6	∝	+	+		
Protein RUFY3	Rufy3	Q5FVJ0	∝	no information			
Protein TANC2	Tanc2	A2A690	∝	no information			
Rabphilin-3A	Rph3a	P47709	∝	+	+		
Ras GTPase-activating protein SynGAP	Syngap1	Q9QUH6	∝	+	+		
Ras-related C3 botulinum toxin substrate 1	Rac1	P63001	∝	+	+	+	
Ras-related protein M-Ras	Mras	O08989	∝	+	+	+	
Ras-related protein Rab-8A	Rab8a	P35280	∝	+	+	+	
Ras-related protein Ral-A	Rala	P63322	∝	+	+	+	
Ras-related protein Rap-1A	Rap1a	P62835	∝	+	+	+	
Regulating synaptic membrane exocytosis protein 1	Rims1	Q9JIR4	1.6	+	+		
Rho GTPase-activating protein 33	Arhgap33	Q80YF9	∝	+	+		
Ribosome-binding protein 1	Rrbp1	Q99PL5	10.0	+			
SAFB-like transcription modulator	Sltm	Q8CH25	∝				
Septin-7	Sept7	O55131	∝	+	+	+	
Serine/arginine repetitive matrix protein 2	Srrm2	Q8BTI8	∝				
Serine/arginine-rich splicing factor 7	Srsf7	Q8BL97	1.8			+	
Serine/threonine-protein kinase DCLK1	Dclk1	Q9JLM8	2.9	no information			
Serine/threonine-protein kinase DCLK2	Dclk2	Q5MPA9	65.0				
Serine/threonine-protein kinase MARK1	Mark1	O08678	8.0	+	+		
Serine/threonine-protein kinase MARK2	Mark2	O08679	11.0	+	+		
Signal recognition particle 54 kDa protein	Srp54	Q6AYB5	∝				
**Sodium/potassium-transporting ATPase subunit alpha-3**	**Atp1a3**	**P06687**	**6.0**	**+**	**+**		+ [18]
Sodium/potassium-transporting ATPase subunit beta-1	Atp1b1	P07340	2.5	+	+	+	+ [18]
SRC kinase signaling inhibitor 1	Srcin1	Q9QXY2	3.2	+	+		
Stress-70 protein, mitochondrial	HSPA9	O35501	∝				+ [16]
Synapsin-1	Syn1	P09951	29.7	+	+		
Synapsin-2	Syn2	Q63537	∝	+	+		
Synaptosomal-associated protein 47	Snap47	Q6P6S0	∝	+	+		
TBC1 domain family member 10B	Tbc1d10b	Q8BHL3	∝	+	+		
T-complex protein 1 subunit alpha	Tcp1	P11983	∝	+	+	+	
T-complex protein 1 subunit beta	Cct2	P80314	∝	+	+	+	
T-complex protein 1 subunit delta	Cct4	P80315	∝			+	
T-complex protein 1 subunit gamma	Cct3	P80318	∝	+	+	+	
T-complex protein 1 subunit zeta	Cct6a	P80317	∝			+	
Testican-1	Spock1	Q62288	∝			+	+ [29]
Thyroid hormone receptor-associated protein 3	Thrap3	Q5M7V8	∝				
Tomoregulin-1	Tmeff1	Q9QYV1	∝	+	+		+ [30]
Traf2 and NCK-interacting protein kinase	Tnik	P83510	∝	+	+	+	
Transformer-2 protein homolog beta	Tra2b	P62996	∝				
Transforming protein RhoA	Rhoa	P61589	∝	+	+		
Trifunctional enzyme subunit alpha, mitochondrial	Hadha	Q64428	∝	+			
Tropomyosin alpha-3 chain	Tpm3	Q63610	∝				
Uncharacterized protein KIAA1107	Kiaa1107	Q80TK0	∝	no information			
Vesicle-associated membrane protein-associated protein A	Vapa	Q9WV55	∝	+	+		
Vimentin	Vim	P31000	32.5				
Voltage-dependent anion-selective channel protein 1	Vdac1	Q60932	5.0	+	+	+	+ [31]
WD repeat-containing protein 37	Wdr37	Q8CBE3	∝	no information			
Zinc finger CCCH domain-containing protein 18	Zc3h18	Q6TQE1	∝				
ZW10 interactor	Zwint	Q8VIL3	∝				

Table 3

List of proteins from whole astrocytes lysates interacting with extracellularly applied oligomeric α-syn

Fold change corresponds to the average spectral count ratio between exposed cells and control cells of three independent replicates. ∞: the spectral count ratio is infinite as the protein is pulled-down only with fibrillarα-synuclein. Membrane and/or extracellular proteins are indicated as determined using the NCBI annotation tool of the Scaffold software based on Gene Ontology (GO) cell component term annotations. References describing extracellular exposure in peer-reviewed articles are indicated.

				GO cell component term annotations from NCBI			Experimental description of extracellular exposure in peer-reviewed articles
Protein name	Gene name	Accession Number	Fold Change (Syn/Ctrl)	Membrane	Plasma membrane	Extracellular region	
14-3-3 protein epsilon	Ywhae	P62260	2.7	+	+	+	
40S ribosomal protein S9	Rps9	P29314	1.8				
60S ribosomal protein L13a	Rpl13a	P35427	1.7	+	+		
60S ribosomal protein L26	Rpl26	P61255	2.7				
60S ribosomal protein L7a	Rpl7a	P62425	2.2	+	+	+	
78 kDa glucose-regulated protein	Hspa5	P06761	4.4	+	+	+	+ [16,17]
Alpha-enolase	Eno1	P04764	1.7				
Arginyl-tRNA synthetase, cytoplasmic	Rars	P40329	∝	+		+	
Aspartyl-tRNA synthetase, cytoplasmic	Dars	P15178	1.9	+		+	
Bifunctional aminoacyl-tRNA synthetase	Eprs	Q8CGC7	1.6	+			
Destrin	Dstn	Q7M0E3	7.0				
Elongation factor 1-delta	Eef1d	Q68FR9	2.0				
FH1/FH2 domain-containing protein 1	Fhod1	Q6P9Q4	3.0	+			
Low-density lipoprotein receptor-related protein 2	Lrp2	P98158	6.5	+	+	+	
Lysyl-tRNA synthetase	Kars	Q99MN1	2.2				
Major vault protein	Mvp	Q62667	7.0			+	
NHP2-like protein 1	Nhp2l1	P55770	3.0				
Polyadenylate-binding protein 1	Pabpc1	Q9EPH8	4.5	+	+	+	
Protein kinase C delta-binding protein	Prkcdbp	Q9Z1H9	2.0	+	+		
Protein lin-7 homolog C	Lin7c	Q792I0	1.6	+	+	+	
Putative ATP-dependent RNA helicase DHX30	Dhx30	Q5BJS0	2.3				
Septin-2	Septin2	Q91Y81	1.7	+	+	+	
SPATS2-like protein	Spats2l	Q5U2T3	2.2				
Stress-70 protein, mitochondrial	Hspa9	P48721	2.5				+ [16]
Tricarboxylate transport protein, mitochondrial	Slc25a1	P32089	2.0	+			

Table 4

List of proteins from whole astrocytes lysates interacting with extracellularly applied fibrillar α-syn

Fold change corresponds to the average spectral count ratio between exposed cells and control cells of three independent replicates. ∞: the spectral count ratio is infinite as the protein is pulled-down only with fibrillarα-synuclein. Membrane and/or extracellular proteins are indicated as determined using the NCBI annotation tool of the Scaffold software based on Gene Ontology (GO) cell component term annotations. References describing extracellular exposure in peer-reviewed articles are indicated.

				GO cell component term annotations from NCBI			Experimental description of extracellular exposure in peer-reviewed articles
Protein name	Gene name	Accession Number	Fold Change (Syn/Ctrl)	Membrane	Plasma membrane	Extracellular region	
2',3'-cyclic-nucleotide 3'-phosphodiesterase	Cnp	P13233	∝	+	+	+	
40S ribosomal protein S15	RPS15	P62842	∝				
40S ribosomal protein S16	Rps16	P14131	2.2	+	+	+	
40S ribosomal protein S17	Rps17	P04644	8.0				
40S ribosomal protein S18	Rps18	P62270	6.2	+	+	+	
40S ribosomal protein S25	Rps25	P62852	4.5			+	
40S ribosomal protein S29	Rps29	P62274	3.0	+	+	+	
40S ribosomal protein S7	Rps7	P62082	2.0	+	+	+	
60S ribosomal protein L22-like 1	Rpl22l1	Q9D7S7	∝				
60S ribosomal protein L24	Rpl24	P83732	1.8	+		+	
60S ribosomal protein L38	Rpl38	P63174	∝				
78 KDa Glucose-regulated protein	Hspa5	P06761	34.0	+	+	+	+ [16,17]
Actin, cytoplasmic	Actb	P60711	∝	+	+	+	
ADP/ATP translocase 2	Slc25a5	P51881	2.3	+	+	+	
ADP-ribosylation factor GTPase-activating protein 2	Arfgap2	Q3MID3	∝	+	+		
ADP-ribosylation factor GTPase-activating protein 3	Arfgap3	Q4KLN7	∝	+			
Alpha-actinin-1	Actn1	Q9Z1P2	∝	+	+	+	
Alpha-crystallin B chain	CRYAB	P05811	∝				
Annexin A1	Anxa1	P07150	2.0	+	+	+	
AP-2 complex subunit mu	Ap2m1	P84091	2.5	+	+	+	
Apolipoprotein E	Apoe	P02650	∝	+	+	+	+ [21]
Ataxin-2	Atxn2	O70305	∝	+			
ATP synthase subunit gamma, mitochondrial	Atp5c1	P35435	15.0	+			
ATP synthase subunit O, mitochondrial	Atp5o	Q06647	∝	+			
ATP-dependent RNA helicase DDX3X	Ddx3x	Q62167	2.9	+		+	
Band 4.1-like protein 5	Epb41l5	Q5FVG2	∝	+	+		
Calmodulin	Calm1	P62161	2.5	+	+	+	
Calmodulin-regulated spectrin-associated protein 2	Camsap2	Q8C1B1	∝				
Calponin-1	Cnn1	Q08091	∝	+	+		
Cdc42 effector protein 1	Cdc42ep1	A1A5P0	∝	+	+		
Cell division control protein 42 homolog	Cdc42	P60766	∝	+	+	+	
Charged multivesicular body protein 2b	Chmp2b	Q8BJF9	∝	+		+	
Chordin-like protein 1	Chrdl1	Q76LD0	9.0			+	
CLIP-associating protein 2	Clasp2	Q99JD4	16.0	+	+		
Cofilin-1	Cfl1	P45592	2.0	+	+	+	
Cytoplasmic dynein 1 heavy chain 1	Dync1h1	Q9JHU4	1.6	+		+	
DnaJ homolog subfamily A member 1	Dnaja1	P63036	∝	+		+	
DnaJ homolog subfamily A member 2	Dnaja2	Q9QYJ0	∝	+		+	
DnaJ homolog subfamily B member 1	Dnajb1	Q9QYJ3	∝			+	
DnaJ homolog subfamily B member 6	Dnajb6	O54946	∝	+			
Elongation factor 1-alpha 1	Eef1a1	P10126	2.0	+	+	+	
Elongation factor Tu, mitochondrial	Tufm	P85834	5.0	+		+	
Filamin-A	Flna	Q8BTM8	1.8	+	+	+	
Friend of PRMT1 protein	Fop	Q9CY57	5.0				
G kinase-anchoring protein 1	Gkap1	Q5XIG5	3.0				
Glial fibrillary acidic protein	Gfap	P47819	3.2	+			
Glutathione peroxidase 1	Gpx1	P04041	2.0			+	
Glypican-4	Gpc4	P51655	∝	+	+	+	+ [24]
Golgi-associated plant pathogenesis-related protein 1	Glipr2	Q9CYL5	7.0	+		+	
Heat shock cognate 71 kDa protein	HSPA8	P63018	3.4	+	+		+ [16]
Heterogeneous nuclear ribonucleoprotein A1	Hnrnpa1	P04256	∝				
Heterogeneous nuclear ribonucleoprotein D0	Hnrnpd	Q60668	∝			+	
Heterogeneous nuclear ribonucleoprotein G retrogene-like	Rbmxrtl	P84586	1.6			+	
Heterogeneous nuclear ribonucleoprotein U-like protein 2	Hnrnpul2	Q00PI9	25.0	+			
Heterogeneous nuclear ribonucleoproteins A2/B1	Hnrnpa2b1	A7VJC2	∝				

				GO cell component term annotations from NCBI			Experimental description of extracellular exposure in peer-reviewed articles
**Protein name**	**Gene name**	**Accession Number**	**Fold Change (Syn/Ctrl)**	**Membrane**	**Plasma membrane**	**Extracellular region**	
Histone H3.3	H3f3b	P84245	2.2	no information			
Histone H1.2	Hist1h1c	P15864	∝				
Histone H2A.Z	H2afz	P0C0S6	∝			+	
IQ motif and SEC7 domain-containing protein 1	Iqsec1	Q8R0S2	∝	+	+		
associated protein 1	Khdrbs1	Q60749	∝	+			
Kinesin-1 heavy chain	Kif5b	Q2PQA9	∝	+			
Lamin-B1	Lmnb1	P70615	∝	+			
Long-chain fatty acid transport protein 1	Slc27a1	P97849	1.6	no information			
MAP7 domain-containing protein 1	Map7d1	A2AJI0	∝				
Microtubule-associated protein 2	Map2	P15146	5.4				
Microtubule-associated protein 4	Map4	Q5M7W5	3.9	+	+	+	
Microtubule-associated protein 6	Map6	Q63560	∝				
Mitochondrial import inner membrane translocase subunit TIM44	Timm44	O35094	∝	+			
Multifunctional protein ADE2	Paics	P51583	3.0	no information			
Myosin regulatory light chain 12B	Myl12b	P18666	3.8				
Myosin-10	Myh10	Q61879	1.6			+	
Nestin	Nes	P21263	∝				
Non-muscle caldesmon	Cald1	Q62736	4.4				
Nuclear pore complex protein Nup214	Nup214	Q80U93	∝	+	+		
Nuclear pore complex protein Nup98-Nup96	Nup98	P49793	∝	+			
PDZ and LIM domain protein 5	Pdlim5	Q62920	19.0	+	+		
PDZ and LIM domain protein 7	Pdlim7	Q3TJD7	6.0	+	+		
Pinin	Pnn	O35691	∝	+	+		
Pleckstrin homology-like domain family B member 1	Phldb1	Q6PDH0	34.0	no information			
Plectin	Plec	P30427	4.8	+	+		
Pre-B-cell leukemia transcription factor-interacting protein 1	Pbxip1	A2VD12	1.8				
Prelamin-A/C	Lmna	P48679	3.3	+			
Protein FAM164A	Fam164a	Q8BJH1	3.0	no information			
Protein lin-7 homolog C	Lin7c	O88952	1.7	+	+	+	
Ras-related C3 botulinum toxin substrate 1	Rac1	P63001	∝	+	+	+	
Ras-related protein Rab-13	Rab13	P35286	∝	+	+	+	
Ras-related protein Rab-8A	Rab8a	P35280	∝	+	+	+	
Ras-related protein Rab-8B	Rab8b	P61028	∝	+	+	+	
Ras-related protein Ral-A	Rala	P63321	∝	+	+	+	
Ras-related protein Rap-1A	Rap1a	P62835	∝	+	+	+	
Rho-related GTP-binding protein RhoG	Rhog	P84096	∝	+	+	+	
Ribosome biogenesis regulatory protein homolog	Rrs1	A1A5P2	3.0				
Septin-7	Sept7	O55131	1.8	+	+	+	
Serine/arginine repetitive matrix protein 2	Srrm2	Q8BTI8	∝				
Serine/threonine-protein kinase DCLK1	Dclk1	Q9JLM8	32.0	no information			
Serine/threonine-protein phosphatase PGAM5, mitochondrial	Pgam5	Q562B5	7.0	+			
Serpin H1 (Heat shock protein 47)	Serpinh1	P19324	6.7			+	
Signal recognition particle 54 kDa protein	Srp54	P14576	∝				
Sorbin and SH3 domain-containing protein 2	Sorbs2	O35413	∝				
Stress-70 protein, mitochondrial	HSPA9	O35501	3.3				+ [16]
Sulfide:quinone oxidoreductase, mitochondrial	Sqrdl	Q9R112	5.5	+			
Trifunctional enzyme subunit alpha, mitochondrial	Hadha	Q64428	4.7	+			
Trifunctional enzyme subunit beta, mitochondrial	Hadhb	Q60587	3.0	+		+	
Vesicle-associated membrane protein-associated protein A	Vapa	Q9WV55	4.0	+	+		
Vimentin	Vim	P31000	2.4				
Vinexin	Sorbs3	Q9R1Z8	∝	+	+		
Voltage-dependent anion-selective channel protein 1	Vdac1	Q60932	∝	+	+	+	+ [31]
Voltage-dependent anion-selective channel protein 2	Vdac2	P81155	∝	+			+ [31]
